# Effects of Partial Replacment of Dietary Forage Using Kelp Powder (*Thallus laminariae*) on Ruminal Fermentation and Lactation Performances of Dairy Cows

**DOI:** 10.3390/ani9100852

**Published:** 2019-10-22

**Authors:** Fuguang Xue, Fuyu Sun, Linshu Jiang, Dengke Hua, Yue Wang, Xuemei Nan, Yiguang Zhao, Benhai Xiong

**Affiliations:** 1 State Key Laboratory of Animal Nutrition, Institute of Animal Science, Chinese Academy of Agricultural Sciences, Beijing 100193, China; xuefuguang123@163.com (F.X.); sfycaas@163.com (F.S.); dengke_h@163.com (D.H.); wangyue9313@163.com (Y.W.); xuemeinan@126.com (X.N.); 2 College of Animal Science and Technology, China Agricultural University, Beijing 100193, China; 3 Beijing Key Laboratory for Dairy Cow Nutrition, Beijing University of Agriculture, Beijing 102206, China; jls@bua.edu.cn

**Keywords:** dairy cows, metagenomic sequencing, kelp powder, rumen fermentation

## Abstract

**Simple Summary:**

Kelp powder has been widely used as feed ingredient or additives in monogastric animal production and presented positive effects on production performance and intestinal microbiota because of its abundance in biomass output, novel oligosaccharides, and iodine. However, little information is available for the nutritional effects of kelp on ruminants. Therefore, the objective of the present study was to investigate the effects of kelp partial replacing dietary forage on rumen fermentation and production performance of dairy cows. Results indicated that, a substitution of 5% kelp powder for forage in the diet reduced the fiber content in the feeding diet and then regulated the proliferation of ruminal microbiota, which lead to a significant reduction of NH_3_-N while increasing acetate, propionate, and total volatile fatty acids (TVFA) concentrations in rumen, and finally partly increasing milk production and milk fat content. The present study may provide more comprehensive cognition of the nutritional value of kelp powder as a dietary raw material. It may further promote the utilization of kelp powder and provide a useful dietary raw material to the husbandry of dairy cows.

**Abstract:**

Background: Kelp powder, which was rich in novel oligosaccharides and iodine might be utilized by the rumen microbiome, promoted the ruminal fermentation and finally enhanced the lactation performance of dairy cows. Therefore, the purpose of this study was to investigate the effects of kelp powder partially replacing dietary forage on rumen fermentation and lactation performance of dairy cows. (2) Methods: In the present study, 20 Chinese Holstein dairy cows were randomly divided into two treatments, a control diet (CON) and a kelp powder replacing diet (Kelp) for a 35-d long trial. Dry matter intake (DMI), milk production, milk quality, ruminal fermentable parameters, and rumen microbiota were measured to investigate the effects of kelp powder feeding on dairy cows. (3) Results: On the lactation performance, kelp significantly increased milk iodine content and effectively enhanced milk production and milk fat content. On the fermentable aspects, kelp significantly raised TVFA while reducing the ammonia-N content. On the rumen microbial aspect, kelp feeding significantly promoted the proliferation of *Firmicutes and Proteobacteria* while suppressing *Bacteroidetes*. (4) Conclusion: kelp powder as an ingredient of feedstuff might promote the rumen fermentation ability and effectively increase milk fat and iodine content, and consequently improve the milk nutritional value.

## 1. Introduction

Kelp powder (*Thallus laminariae*), attracts more attentions in recent years in the husbandry because of its high-yielding biomass and abundant nutritional content such as crude protein, amino acids, polyunsaturated fatty acids, and oligosaccharides [1,2]. Kelp has been used as the feed ingredient or feed additive in the production of monogastric animals such as layers, broilers, and pigs, and expressed positive effects on animal performance, such as reducing the incidence of diseases and promoting production performance [3,4,5,6]. Whereas little information is available on kelp nutritional effects on ruminants, while it has been found that that kelp may potentially lower somatic cell count and improve body condition of dairy cattle [7]. 

Ruminants have high digestibility of feeding ingredients for the ruminal microbial ecosystem comprised of an immense variety of microbiota which execute the complex anaerobic metabolism [8]. Bacteria in the rumen contribute more than 95% of the whole biomass and perform the main function in rumen fermentation [9,10]. For this purpose, oligosaccharides which were not degraded in monogastric creatures could be degraded in the rumen and hydrolyzed into mono- or di- saccharides and produced formate, acetate, and lactate as the end products [11]. Moreover, ruminal microbiota might be altered for the increasing ruminal end products and so might the fermentation procedure. Finally, the production performance might be influenced. 

Therefore, we hypothesized that oligosaccharides in kelp will be digested in rumen, increase the volatile fatty acids (VFAs) content, and finally promote the lactation performance of ruminants. The objective of the present study was to investigate the effects of partially replacing dietary forage using kelp powder on ruminal microbiota, ruminal fermentation parameters, and the lactation performance of dairy cows.

## 2. Materials and Methods 

All experimental protocols performed in this study were approved by the Animal Ethics Committee of the Chinese Academy of Agricultural Sciences (Beijing, China). The experimental procedures used in this study were in accordance with the recommendations of the academy’s guidelines for animal research.

### 2.1. Kelp Powder

The kelp powder (brown algae, purity 100%, Wanfang Biological Co., Ltd., Xi’an, China) used in the current study was harvested in Weihai city, Shandong province, China (37°16′ N, 122°41′ E) in July 2017. It was then dried and milled as a gray-green powder. The chemical composition of the kelp powder is shown in Table S1.

### 2.2. Animals and Experimental Design

A completely randomized design was applied in the present study. Twenty Chinese Holstein lactating dairy cows (589 ± 19.9 kg BW; 160 ± 18 DIM, milk yield (22 ± 2.3 kg/day)) were randomly divided into two treatments. Treatments included a control diet (CON) and a kelp powder replacing diet (Kelp). In the kelp treatment, kelp was used partly replace corn silage, oat hay and fatty acid calcium which resulted in kelp content came to 5% (dry matter (DM) basis). The diets were formulated according to NRC (2001) to meet or exceed the energy requirements of Holstein dairy cows yielding 20 kg of milk/day with 3.5% milk fat and 3.0% true protein. Details of ingredient analysis and chemical composition of diets were shown in Table 1.

### 2.3. Feeding Management

All cows were raised in individual stalls of a 35 d-long period feeding procedure. The cows were fed three times a day (07:00, 13:00 and 18:00 h, respectively) ad libitum in order to ensure that the total mixed ration (TMR) were fresh and available for the cows for at least 20 h a day. Cows were milked three times a day (09:00, 15:00, and 20:00 h, respectively) and were managed with natural lighting. All cows were dewormed before the commencement of this study. 

### 2.4. Sampling and Parameters Measurement

During the experimental period, automatic feeding equipment (made by Institute of Animal Science Chinese Academy of Agricultural Sciences, Beijing, China and NanShang Husbandry Science and Technology Ltd. Henan, China) was used to record dry matter intake. Milking facilities (90 Side-by-Side Parallel Stall Construction, Afimilk, Israel) were applied to record milk production of each cow. 

On the last day of the experiment, a gastric rumen sampler was used to collect rumen fluid samples through the esophagus at 3 h after the morning feeding. Collected samples were strained through four layers of cheesecloth to obtain rumen fluid. Rumen fluid was then divided into two parts. One part was processed to analyze the pH value, rumen volatile fatty acid (VFAs) and ammonia-N (NH_3_-N). The other part was put into the liquid nitrogen immediately after adding stabilizer and then stored at −80 °C for DNA extraction. Rumen contents were strained through four layers of cheesecloth with a mesh size of 250 μm. The pH of each rumen fluid sample was measured immediately using a portable pH meter (Testo 205, Testo AG, Lenzkirch, Germany). Individual and total VFAs (TVFA) in the aliquots of ruminal fluid were determined by gas chromatograph (GC-2010, Shimadzu, Kyoto, Japan). Concentration of NH_3_-N was determined by indophenol method and the absorbance value was measured through UV-2600 ultraviolet spectrophotometer (Tianmei Ltd., China).

Milk samples were collected from individual cows during the last four consecutive days in 100-mL vials in each milking period. Samples were preserved with 2-bromo-2-nitropropan-1,3-diol and stored at 4 °C, before sent to the Milk and Dairy Products Quality Supervision and Testing Center, Ministry of Agriculture (Beijing, China) for analyses of milk protein, fat, lactose, somatic cell count and milk iodine content by a mid-infrared spectroscopy (Fossomatic 4000, Foss Electric A/S, Hillerød, Denmark).

### 2.5. DNA Extraction and Sequencing Process

DNA for metagenomics sequencing was extracted from the rumen fluid samples by using the QIAamp DNA Stool Mini Kit (Qiagen, Hilden, Germany) according to manufacturer’s protocols. The DNA concentration and purity were quantified with TBS-380 and NanoDrop2000, respectively. DNA quality was examined with the 1% agarose gels electrophoresis system.

The bacteria 16S rRNA gene was amplified using the barcoded universal primers 338F (5′-barcorde-ACTCCTRCGGGAGGCAGCAG-3) and 806R (5′-GGACTACCVGGGTATCTAAT-3′) spanning the V3–V4 hyper variable region. The 16s RNA sequencing procedure was conducted through Illumina HiSeq 4000 platform (Illumina Inc., San Diego, CA, USA). Quality filtering on the raw tags were performed under specific filtering conditions to obtain the high-quality clean tags according to the QIIME (V1.7.0) quality-controlled process. The tags were compared with the reference database using UCHIME algorithm to detect chimera sequences, and then the chimera sequences were removed. Then the effective tags were finally obtained. Sequences analysis were performed by Uparse software (Uparse v7.0.1001, Tiburon, California, USA). Sequences with >97% similarity were assigned to the same OTUs. A representative sequence for each OTU was screened for further annotation. For each representative sequence, the GreenGene Database was used based on RDP classifier algorithm to annotate taxonomic information. Abundance information of OTUs was normalized using a standard of sequence number corresponding to the sample with the least sequences. Subsequent analysis of alpha diversity and beta diversity were all performed basing on this output normalized data. Alpha diversity is applied in analyzing complexity of species diversity for a sample through six indices, including, Chao1, Shannon, Simpson, ACE. All indices in our samples were calculated with QIIME (Version 1.7.0) and displayed with R software (Version 3.3.1, R Core Team, Vienna, Austria)). Beta diversity analysis was used to evaluate differences of samples in species complexity. Beta diversity on unweighted unifrac were calculated by QIIME software (Version 1.7.0, San Diego, CA, USA). Cluster analysis was preceded by principal coordinates analysis (PCoA), which was applied to reduce the dimension of the original variables. PCoA analysis was displayed by WGCNA package, stat packages, and ggplot2 package in R software. Unweighted Pair-Group Method with Arithmetic Means (UPGMA) Clustering was performed as a type of hierarchical clustering method to interpret the distance matrix using average linkage and was conducted by QIIME.

### 2.6. Statistical Analysis 

Results were presented as means ± SEM. A normal distribution test of ruminal pH, VFAs, and NH_3_-N were first conducted using SAS procedure “proc univariate data = test_normal” and then Student’s *t*-test of SAS 9.2 was applied to analyze the differences of parameters between CON and Kelp treatments. *p*-value < 0.05 was considered to be significance and 0.05 ≤ *p* < 0.10 was considered as a tendency. Barplot, principal coordinate analysis (PCoA), hierarchical clustering analysis (HCA) for different rumen bacteria were conducted using R package. Spearman correlations between bacteria communities and ruminal fermentation variables were assessed using the PROC CORR procedure of SAS 9.2 (SAS Institute, Inc., Cary, NC, USA). Relative abundance of all phyla of bacteria were chosen to conduct the correlation analysis. A correlation matrix was created and visualized in a heatmap format using R package version 3.3.1. 

## 3. Results

### 3.1. Animal Production Performance and Rumen Fermentation Parameters

The animal production performance was the most concerned in the dairy cattle production. In the present study, as shown in Table 2, the DMI, milk production, milk fat increased after kelp feeding, however, not significantly. Particularly, the milk iodine content significantly increased in the kelp treatment compared with CON treatment (*p* < 0.05). On the aspect of fermentation parameters, the kelp significantly raised ruminal total VFAs content while reduced the ammonia-N content (*p* < 0.05). For the individual VFA, kelp feeding significantly increased ruminal acetate, propionate and isobutyrate content.

### 3.2. Sequencing Information

The bacteria 16S rRNA gene was amplified using the barcoded universal primers 338F and 806R spanning the V3–V4 hyper variable region. The sequencing reads number of each samples were between 30,000–45,000 and the mean length of each reads were more than 430 nt. All sequencing information has been shown in Additional File 2. Sequences with > 97% similarity were assigned to the same OTUs and then aligned to GreenGene Database based on RDP classifier algorithm to annotate taxonomic information. Totally, 1338 OTUs, 17 phyla and more than 220 genera were identified in the present study, and all the taxonomy information is displayed in Additional File 3. 

### 3.3. Effects of Kelp Replacing Diet on Ruminal Bacteria

#### 3.3.1. α-Diversity

All identified bacteria were chosen for further analysis to investigate the effects of kelp replacing diet on the community of ruminal bacteria. Alpha diversity is applied in analyzing complexity of species diversity for a sample through Chao1, Shannon, Simpson, ACE. All these indices of α-diversity were displayed in Table 3. Based on the results, no significant changes were found in the kelp treatment compared with CON.

#### 3.3.2. β-Diversity

**Figure 1:**
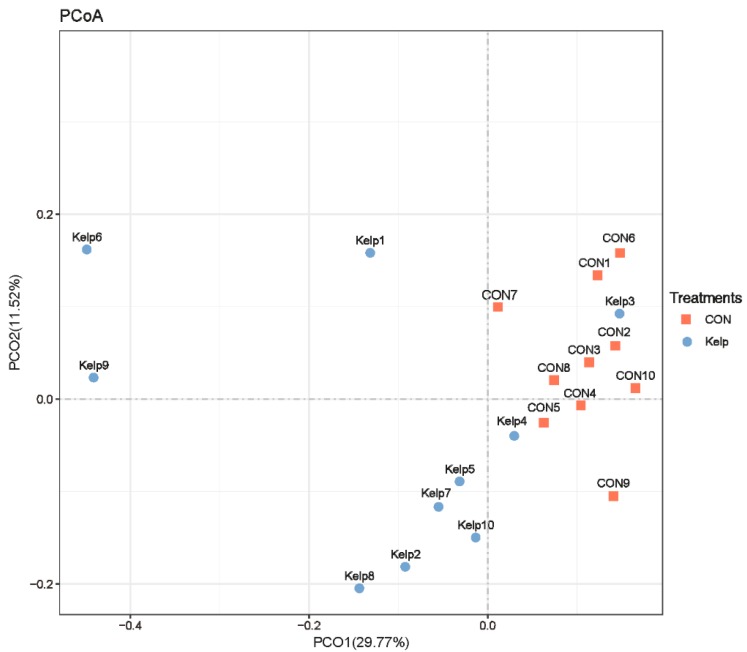
Principal coordinate analysis (PCoA) of bacteria community structures in CON and kelp treatment. CON = control diet; Kelp = kelp powder replacing diet.

Beta diversity analysis was then conducted to evaluate differences of samples in species complexity. PCoA based on unweighted UniFrac distance metrics was conducted to compare bacterial profile among the three treatments. As shown in Figure 1, PCoA axes 1 and 2 accounted for 29.77% and 11.52% of the total variation, respectively. Based on the results, bacteria community in kelp treatment could be separated from those in the CON by PCo1 except Kelp3.

Since the kelp made a difference to the whole bacteria community, a differential analysis on ruminal bacteria in different levels was then conducted to investigate the effects of kelp on the abundances of ruminal bacteria. Results are shown in Table 4 and Table 5, in the level of phyla and genera, respectively. Based on the Table 4, *Bacteroidetes*, *Firmicutes,* and *Proteobacteria* contribute the three most abundant phyla of ruminal bacteria and the kelp feeding significantly promoted the proliferation of *Firmicutes* and *Proteobacteria* while suppressing *Bacteroidetes*. No significant effects were found of kelp feeding on other phyla. At the genera level, as shown in Table 5, *Prevotella*, *Ruminococcaceae*, *Ruminococcus*, *Bacteroidales*, *Prevotellaceae*, *Succinivibrionaceae*, *Lachnospiraceae*, and *Succiniclasticum* were the most abundant genera in the present study. *Prevotella*, *Bacteroidale*, *Rikenellacea*, *Selenomonas*, and *Saccharofermentans* were significantly decreased while *Ruminococcaceae*, *Ruminococcus*, *Lachnospiraceae*, and *Sharpea* were significantly increased after kelp feeding. There were no significant changes of other genera. 

**Figure 2:**
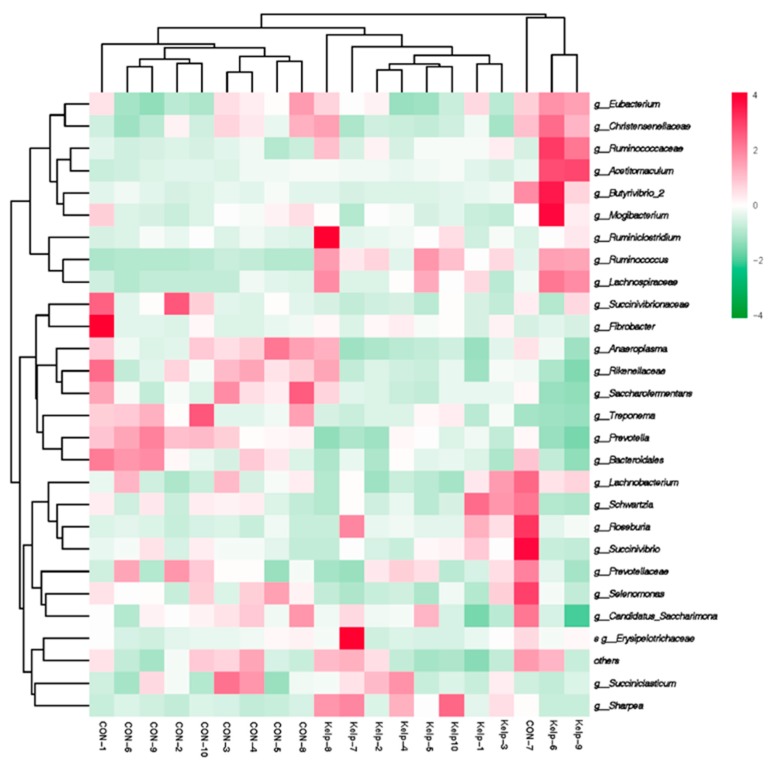
Hierarchical clustering analysis (HCA) and heat map analysis on abundances of ruminal bacteria content between CON and kelp treatment on the level of genera. Rows represent genera of bacteria and columns represent samples. Cells were colored based on the relative abundance of bacteria measured in rumen, red represents high rumen levels while green represents low signal intensity and white cells showing the intermediate level.

Hierarchical clustering analysis (HCA) and heat map analysis was conducted to further understand the effects of kelp feeding on ruminal bacteria profile. Results of HCA analysis are displayed in Figure 2. Bacteria which belongs to CON and kelp treatments could separate into two big clusters clearly except CON7. Ruminal bacteria in genera level gathered into two big clusters. The genera in upper half part were increased after kelp feeding which consists of *g__Eubacterium*, *g__Christensenellaceae*, *g__Ruminococcaceae*, *g__Acetitomaculum*, *g__Butyrivibrio*, and *g__Mogibacterium.* Lower half part consists of those genera which were decreased after kelp feeding, such as *g__Succinivibrionaceae*, *g__Fibrobacter*, *g__Anaeroplasma*, *g__Rikenellaceae*, and *g__Saccharofermentans*. 

### 3.4. Correlations between Bacteria Communities and Ruminal Fermentation Parameters 

**Figure 3:**
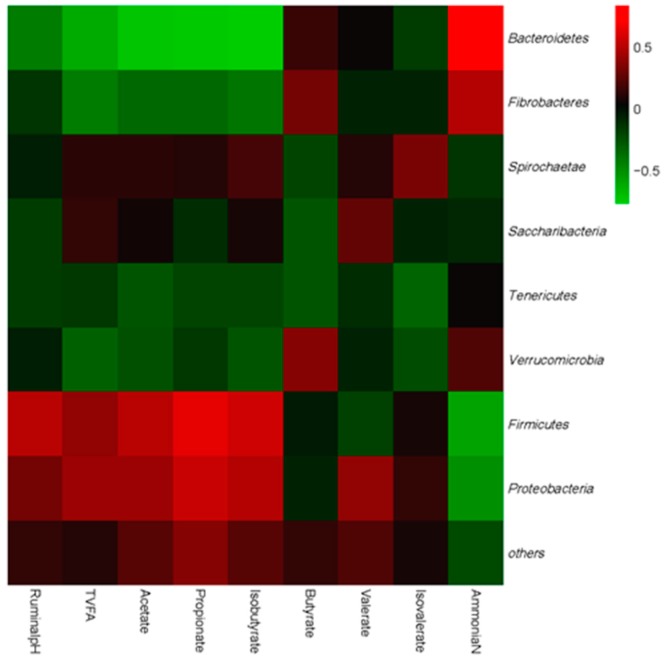
Correlation analyses between abundances of ruminal bacteria and DMI, milk quality parameters, and ruminal fermentation parameters on the level of phyla. The red color represents a positive correlation while the green color represents a negative correlation.

**Figure 4:**
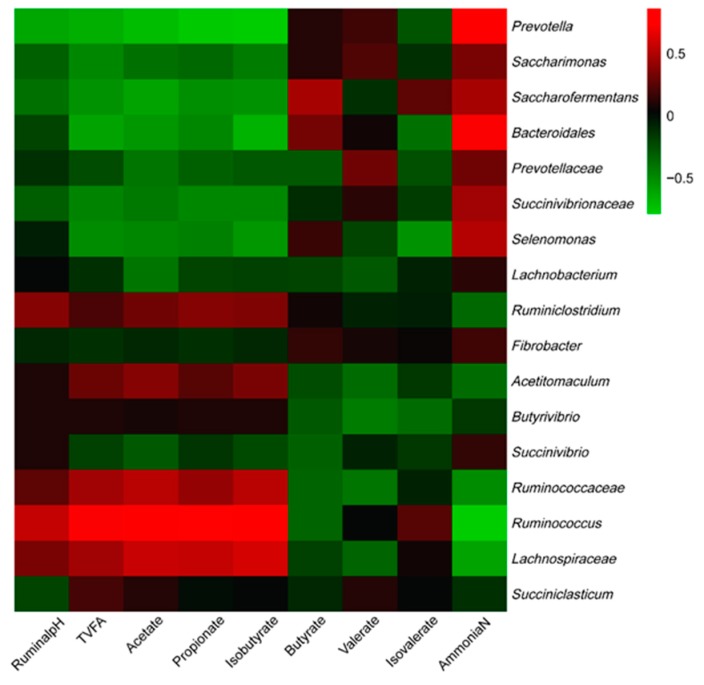
Correlation analyses between the most abundant genera of ruminal bacteria and DMI, milk quality parameters, and ruminal fermentation parameters. The red color represents a positive correlation while the green color represents a negative correlation.

At last, all phyla and most abundant genera were selected to conduct the correlation analysis between bacteria and fermentation parameters for the purpose to investigate the effects of ruminal bacteria on ruminal fermentation. Results are shown in Figure 3 and Figure 4, respectively. All phyla could be separated into two parts based on the correlations with ruminal fermentation parameters. One was positively correlated with ruminal pH and VFA content, while negatively correlated with NH_3_-N, this part was mainly comprised by *Firmicutes* and *Proteobacteria*.

The other part was just conversed, which was positive with NH_3_-N while negative correlated with ruminal VFAs content. This part mainly consisted of *Bacteroidetes*, *Fibrobacteres*, and *Saccharibacteria*. As to the genera level, bacteria could also separate into two big clusters. The first part, which consists of *Ruminiclostridium*, *Acetitomaculum*, *Butyrivibrio*, *Ruminococcaceae*, *Ruminococcus*, *Lachnospiraceae*, and *Succiniclasticum* was positively correlated with pH, TVFAs, acetate, propionate, and Isobutyrate while being negatively correlated with NH_3_-N. The other part was mainly comprised by *Prevotella*, *Saccharimonas*, *Saccharofermentans*, *Bacteroidales*, *Prevotellaceae*, *Succinivibrionaceae*, and *Selenomonas* which performed conversed correlation with the fermentation parameters compared with the first part. 

## 4. Discussion

### 4.1. Effects of Kelp Feeding on Ruminal Carbohydrate Metabolism 

In the present study, ruminal TVFA, acetate and propionate significantly increased after kelp powder diet feeding, which indicated that kelp promoted ruminal carbohydrates metabolism. In ruminal conditions, carbohydrates were always degraded into mono- or di-saccharides, and then hydrolyzed into VFAs [12]. The kelp was rich in oligosaccharides, which are always undegradable in monogastrics, which could be fermented by ruminal microbiota [11]. Because of the lower molecular weight and the simpler structure [13], oligosaccharides might be easier digestible than the polysaccharides such as cellulose. When replacing dietary forage with kelp powder, more carbohydrates were digested in the kelp feeding diets, and therefore, more VFAs were synthesized. 

Kelp treatment significantly decreased the ratio of acetate/propionate. In ruminal conditions, when dietary high-fermentable content increased, it always decreased the ratio of acetate/propionate [14]. In the present study, kelp feeding diets partially decreased the fiber content, which might lead to the reduction of the mainly fiber-degrading bacteria such as *Bacteroidetes* and *Fibrobacteres* [15,16]. Besides, the kelp was added through powder form, which significantly reduced the particle size compared with their substitution, decreasing the retention time in rumen and further reducing the reaction time between fiber-degrading bacteria and dietary fiber [17].

Inversely, the easier digestible oligosaccharides help increase the abundances of *Firmicutes* and *Proteobacteria* which mainly degrade the highly-fermentable carbohydrates in ruminal conditions [18,19]. For the reasons given above, kelp feeding diets contain more highly-fermentable carbohydrate and might lead to accumulation of glucose. In ruminal conditions, glucose digested into pyruvate through glycolytic pathway [20], and then hydrolyzed into acetyl-CoA, which was the precursor of acetate with the help of pyruvate formate-lyase (PFL) [21]. When provided more high-fermentable carbohydrate, pyruvate accumulated and then turn to form more lactate, oxaloacetic acid [22], and further developed into propionate. This might contribute to the significant decrease of acetate/propionate.

### 4.2. Effects of Kelp Feeding on Ruminal Nitrogen Metabolism

Apart from the carbohydrate metabolism, the average rumen NH_3_-N concentration in the kelp feeding treatment was significantly decreased compared with that in the control treatment, which indicated that kelp might affect the ruminal nitrogen(N) metabolism. The ruminal NH_3_-N concentration could be influenced by absorption of rumen wall, ruminal outflow rate, and rumen microbial community [23]. Previous study reported that the ruminal outflow rate was negatively associated with forage length in the diet [24], the longer forage particles reduced the contact surface area with the microorganisms, resulting in a lower synthesis efficiency of rumen microbial protein and consequently increased the NH_3_-N concentration [25]. In the present study, kelp was added through powder form which help increase attached area for ruminal microbiota, thereafter leading to the decrease of ruminal NH_3_-N concentration in kelp feeding.

Pisulewski et al. (2010) obtained best results of ruminal NH_3_-N utilization content with 88–133 mg/L [26], which opportunely included the content in kelp treatment. In ruminal conditions, ruminal bacteria could utilize NH_3_-N to synthesize amino acids, and then form bacteria proteins. For synthesis of some amino acids, specific carbon sources are required, such as isobutyrate for valine [27]. In the present study, we observed that isobutyrate was significantly increased after kelp feeding. The increased isobutyrate might enhance the activity of ruminal bacteria to utilize more NH_3_-N. Therefore, NH_3_-N significantly decreased after kelp feeding. 

In addition, utilization of ruminal NH_3_-N into the bacterial protein in the rumen is energy dependent, providing adequate ruminal available energy is requirement for ruminal NH_3_-N utilization [28]. In ruminal conditions, available energy was mainly provided by VFAs [29], which were found to significantly increase in kelp treatment. Therefore, the activity of rumen microorganisms will be enhanced by the provided energy, and more NH_3_-N could be incorpoarated into bacteria protein.

### 4.3. Effect of Kelp Feeding on Milk Performance

Although not significantly, the milk production and milk fat percentage were increased after kelp feeding to some extent in the present study. Milk fat was a necessary parameter of milk quality and benefits to the human health [30]. In the current study, kelp feeding treatment could effectively promote the milk fat percentage through improving ruminal acetate content [31]. Increasing the replacement percentage of dietary forage with kelp might lead to increasing milk fat, consequenty improving the benefits of milk to human health.

Milk iodine content significantly increased in the present study, which was in line with Kaufmann [32]. For ruminants, iodine deficiency would result in low thyroid hormone concentrations and prolonged gestation [33]. The abundant iodine content in kelp might contribute to the partial increase of milk production.

## 5. Conclusions

In summary, a substitution of 5% kelp powder for forage in the diet reduced the fiber content in the feeding diet and then regulated the proliferation of ruminal microbiota, which lead to a significantly reduced NH_3_-N content while increasing acetate and TVFA concentrations in the rumen. This result indicated that kelp powder as a dietary raw material might promoted the rumen fermentation ability and then benefit the lactation performance of dairy cows. Moreover, it could effectively increase milk fat and iodine content and consequently improve milk’s nutritional value.

## Figures and Tables

**Table 1:** Ingredients and chemical composition of the diets (DM basis) ^A,B^.

Items	Kelp	Control
**Ingredients (%)**		
Corn silage	27.0	29.1
Oat hay	20.4	22.8
Kelp powder	5.0	—
Alfalfa haylage	7.2	7.2
Ground corn	3.6	3.6
Extruded soybeans	2.6	2.6
Soybean meal	9.9	9.9
Rapeseed meal	4.5	4.5
Cottonseed meal	2.6	2.6
Pressed corn	2.2	2.2
Cottonseed	2.7	2.7
Corn hull	9.6	9.6
Fat powder	1.2	1.2
Fatty acid calcium	1.2	1.7
Vitamin/mineral premix	0.3	0.3
**Nutrient composition (%, unless otherwise stated)**		
NE_L_ (Mcal/kg)	1.40	1.41
CP	12.81	12.96
EE	3.42	3.57
NDF	36.78	38.14
ADF	20.69	22.15

^A^ Nutrient composition of the experimental diets were calculated according to NRC (2001); 0% Kelp = no kelp powder in the diet; 5% Kelp = 5% kelp powder in the diet; EE = ether extract; NE_L_ = net energy for lactation and calculated according to NRC (2001). ^B^ Vitamin/mineral premix contained (DM basis): 15.7% of Ca, 4.1% of P, 1600 mg/kg of Fe, 700 mg/kg of Cu, 3500 mg/kg of Mn, 7500 mg/kg of Zn, 80 mg/kg of Se, 400 mg/kg of I, 50 mg/kg of Co, 190,000 IU/kg of vitamin A, 55,000 IU/kg of vitamin D and 1900 IU/kg of vitamin E. NDF = neutral detergent fiber, ADF = acid detergent fiber.

**Table 2:** Effects of kelp powder on animal productivity and ruminal fermentation.

Items	CON	Kelp	SEM	*p*-Value
DMI (kg/day)	16.2	16.6	0.468	0.114
Milk production (kg/day)	21.1	22.7	1.894	0.235
Milk fat (%)	3.8	3.9	0.126	0.146
Milk protein(%)	3.41	3.42	0.061	0.106
Milk iodine(mg/L)	0.07 ^b^	0.12 ^a^	0.020	0.029
Ruminal pH	6.45	6.38	0.194	0.216
Ammonia-N (mg/dL)	17.8 ^a^	13.1 ^b^	1.933	<0.001
Acetate (mmol/L)	65.7 ^b^	67.1 ^a^	0.773	0.038
Propionate (mmol/L)	17.2 ^b^	19.2 ^a^	0.632	0.027
A/P	3.83	3.57	0.098	0.001
Butyrate (mmol/L)	16.3	16.1	0.137	0.356
Isobutyrate (mmol/L)	1.6 ^b^	3.9 ^a^	0.115	0.041
Valerate(mmol/L)	2.21	2.14	0.064	0.241
Isovalerate (mmol/L)	2.31	2.50	0.160	0.216
TVFA (mmol/L)	104.5 ^b^	110.8 ^a^	1.836	0.016

^a,b^ means within a row with different letters differed significantly (*p* < 0.05); SEM = standard error of the mean. A/P = acetate/propionate.

**Table 3:** Effects of kelp powder on α-abundance of ruminal microbiota.

Items	CON	Kelp	FC	Log 2 FC	SE	*p*-Value
ACE	1182.5	1170.2	0.99	−0.02	0.204	0.853
Chao1	1192.4	1189.9	1.00	0.00	0.076	0.97
Shannon	5.42	5.31	0.98	−0.03	0.08	0.398
Simpson	0.014	0.018	1.29	0.36	0.009	0.216
SOBs	1035.3	1016	0.98	−0.03	0.12	0.759

Note, FC = fold change, SE = standard error of the mean.

**Table 4:** Effects of kelp powder on the abundances of ruminal microbiota in phyla level.

Items	CON	Kelp	FC	log2 FC	SE	*p*-Value
*p__Bacteroidetes*	14570 ^a^	10110 ^b^	0.69	−0.53	0.10	<0.001
*p__Firmicutes*	7007 ^b^	12098 ^a^	1.73	0.79	0.13	0.008
*p__Fibrobacteres*	91.4	36.8	0.40	−1.31	0.46	0.088
*p__Proteobacteria*	424.6 ^b^	1787.3 ^a^	4.21	2.07	0.53	0.024
*p__Spirochaetae*	179.3	242.1	1.35	0.43	0.32	0.408
*p__Saccharibacteria*	133.5	143	1.07	0.099	0.32	0.765
*p__Tenericutes*	264	214.4	0.81	−0.30	0.52	0.462
*p__Verrucomicrobia*	33.8	19	0.56	−0.83	0.31	0.277
others	39.4	54.2	1.38	0.46	0.27	0.286

^a,b^ means within a row, different letters differed significantly (*p* < 0.05); SE = standard error.

**Table 5:** Effects of kelp powder on the abundances of ruminal microbiota in genera level.

Items	CON	Kelp	FC	Log2 FC	SE	*p*-Value
*g__Prevotella*	9897.2 ^a^	6376.8 ^b^	0.64	−0.63	0.08	<0.001
*g__Ruminococcaceae*	1470.3 ^b^	3111.1 ^a^	2.12	1.08	0.17	0.021
*g__Ruminococcus*	539.8 ^b^	2603.8 ^a^	4.82	2.27	0.25	0.004
*g__Bacteroidales*	1969.7 ^a^	1064.1 ^b^	0.54	−0.89	0.15	0.002
*g__Prevotellaceae*	1472	1199.2	0.81	−0.30	0.15	0.185
*g__Succinivibrionaceae*	636.3	1238.8	1.95	0.96	0.56	0.247
*g__Lachnospiraceae*	566.4 ^b^	1069.2 ^a^	1.89	0.92	0.15	0.008
*g__Succiniclasticum*	626.6	653.8	1.04	0.06	0.33	0.876
*g__Eubacterium*	502.7	521.8	1.04	0.05	0.27	0.767
*g__Rikenellaceae*	600.5 ^a^	315.6 ^b^	0.53	−0.93	0.13	0.016
*g__Christensenellaceae*	430.8	428.6	0.99	−0.01	0.28	0.986
*g__Selenomonas*	437.1	240.3	0.55	−0.86	0.28	0.012
*g__Acetitomaculum*	171.7	446.2	2.60	1.38	0.18	0.121
*g__Butyrivibrio*	177.1	255.1	1.44	0.53	0.31	0.615
*g__Treponema*	285.8^a^	124 ^b^	0.43	−1.20	0.42	0.022
*g__Erysipelotrichaceae*	140.8	204.2	1.45	0.54	0.51	0.539
*g__Anaeroplasma*	214.9 ^a^	113.3 ^b^	0.53	−0.92	0.35	0.003
*g__Lachnobacterium*	170.3	143.4	0.84	−0.25	0.18	0.453
*g__Candidatus Saccharimonas*	169.6	111	0.65	−0.61	0.22	0.05
*g__Saccharofermentans*	146.9 ^a^	81.2 ^b^	0.55	−0.86	0.31	0.016
*g__Sharpea*	31.2 ^b^	189.7 ^a^	6.08	2.60	0.22	0.016
*g__Roseburia*	84.6	123.2	1.46	0.54	0.6	0.535
*g__Ruminiclostridium*	50.5	150.9	2.99	1.58	0.49	0.159
*g__Schwartzia*	93.5	77.5	0.83	−0.27	0.33	0.642
*g__Lachnospiraceae*	79.8	81.7	1.02	0.03	0.41	0.868
*g__Fibrobacter*	77.3	52.9	0.68	−0.55	0.14	0.654
*g__Mogibacterium*	58	64.9	1.12	0.16	0.53	0.768
*g__Succinivibrio*	60	34	0.57	−0.82	0.28	0.338
others	3665.1	3447.5	0.94	−0.088	0.48	0.471

^a,b^ means within a row, different letters differed significantly (*p* < 0.05); SE = standard error.

## Supplementary Materials

The following are available online at https://www.mdpi.com/2076-2615/9/10/852/s1, Table S1: Chemical composition of kelp powder; Table S2: Sequencing information of each samples; Table S3: Taxonomy results of ruminal bacteria of each samples.
