# Supplementary material for: Effects of Partial Replacment of Dietary Forage Using Kelp Powder (Thallus laminariae) on Ruminal Fermentation and Lactation Performances of Dairy Cows
Source: Animals (Basel). 2019 Oct 22;9(10):852. doi: 10.3390/ani9100852 (PMC6826980; doi:10.3390/ani9100852)
Supplement: Supplementary file 1 [file animals-09-00852-s001.zip › Table S1.docx]

**TABLE 1** Chemical composition of kelp powder

| Chemical composition | Kelp powder (% of DM) | |
| --- | --- | --- |
| DM (% of fresh) | | 91.8 |
| CP | | 6.1 |
| NDF | | 19.8 |
| EE | | 2.9 |
| Ash | | 24.3 |
| Ca | | 2.0 |
| P | | 0.1 |
| K | | 2.4 |
| Mg | | 0.8 |
| I | | 0.4 |
| Mannitol | | 12.5 |
| Alginate | | 26.1 |
